# The Psychological and Behavioural Correlates of Workplace Victimization

**DOI:** 10.3390/brainsci16050544

**Published:** 2026-05-21

**Authors:** Amelia Rizzo, Maria Grazia Maggio, Martina Barbera, Francesca Bruno, Gabriele Giorgi, Luca Di Giampaolo, Murat Yildirim, Lucasz Szarpak, Giuseppe Ferrari, Raffaela Maione, Rocco Salvatore Calabrò, Francesco Chirico

**Affiliations:** 1 Medical Legal Centre of Messina, National Institute of Social Welfare, 98122 Messina, Italy; amrizzo@unime.it; 2 IRCCS Centro Neurolesi Bonino-Pulejo, S.S. 113 Via Palermo, C. da Casazza, 98124 Messina, Italy; raffaela.maione@irccsme.it (R.M.); rocccos.calabro@irccsme.it (R.S.C.); 3 Department of Cognitive Sciences, Pedagogical Psychological and Cultural Studies, University of Messina, 98122 Messina, Italy; martinabarbera17@gmail.com (M.B.); francescabruno.psi@outlook.it (F.B.); 4 Università Europea di Roma, Via Degli Aldobrandeschi 190, 00163 Rome, Italy; gabriele.giorgi@unier.it; 5 Department of Innovative Technologies in Medicine & Dentistry, University “G. d’Annunzio” of Chieti–Pescara, Via dei Vestini, Campus Universitario, 66100 Chieti, Italy; luca.digiampaolo@unich.it; 6 Department of Psychology, Faculty of Science and Letters, Ağrı İbrahim Çeçen University, Fırat Mahallesi Yeni Üniversite Caddesi No: 2 AE/1, Merkez, Ağrı 04100, Türkiye; muratyildirim@agri.edu.tr; 7 Psychology Research Center, Khazar University, 11 Mahsati Ganjavi Rd, Baku 1096, Azerbaijan; 8 Institute of Medical Science, Collegium Medicum, The John Paul II Catholic University of Lublin, 20-708 Lublin, Poland; lukasz.szarpak@gmail.com; 9 Research Unit, Maria Sklodowska-Curie Bialystok Oncology Center, 15-027 Bialystok, Poland; 10 Henry J.N. Taub Department of Emergency Medicine, Baylor College of Medicine, Houston, TX 77030, USA; 11 Italian Society of Integrated Psychotherapy for Social Development, 20129 Milan, Italy; ferrari@sipiss.it; 12 Post-Graduate School of Occupational Health, Università Cattolica del Sacro Cuore, Largo Agostino Gemelli 8, 00168 Rome, Italy; francesco.chirico@unicatti.it; 13 Centro Sanitario Polifunzionale, Health Service Department, Italian State Police, Ministry of the Interior, 00184 Rome, Italy

**Keywords:** workplace victimization, workplace bullying, psychological distress, personality traits, mood states, defense mechanisms, coping strategies, psychometric assessment

## Abstract

**Highlights:**

**What are the main findings?**

- In this sample of adults referred for psychological evaluation in the context of suspected or documented workplace victimization, a coherent psychological profile was observed, characterized by depressive symptoms, somatic complaints, reduced vigor, Anger–Hostility, and paranoid traits across personality (MMPI-2) and mood (POMS) domains.
- Defense mechanisms analysis revealed a predominance of reversal (e.g., altruism and idealization), suggesting a tendency toward internalized coping strategies that may contribute to the maintenance of psychological distress under chronic workplace stress conditions.

**What are the implications of the main findings?**

- The findings indicate that workplace victimization is characterized by a multidimensional convergence of emotional, somatic, and personality-related processes, highlighting the importance of integrated assessments that jointly consider symptom expression and coping mechanisms.
- The predominance of internalizing defense strategies suggests potential targets for clinical intervention focused on emotional regulation and coping flexibility, while future studies should adopt longitudinal and controlled designs to clarify the directionality and specificity of these associations.

**Abstract:**

**Background**: Workplace victimization is a form of repeated and systematic psychological violence that can severely affect both mental and physical health. From a psychological perspective, it impacts mood states, defense mechanisms, and personality functioning. **Methods**: This cross-sectional study investigated the psychological and behavioural correlates of workplace victimization in a sample of 33 workers from various professional sectors, using a multidimensional assessment including standardized measures of personality traits, mood states, and defense mechanisms. **Results**: The MMPI-2 profile revealed elevated scores in Hypochondriasis (Hs: 72.00), Depression (D: 70.21), Hysteria (Hy: 67.61), and Paranoia (Pa: 68.76), indicating somatic symptoms, depressive features, and suspiciousness. The POMS showed increased Tension–Anxiety (T: 65.06), Depression–Dejection (D: 68.21), Anger–Hostility (A: 68.15), and Fatigue–Inertia (F: 65.24), alongside reduced Vigor–Activity (V: 43.18). The DMI analysis highlighted a high Reversal score (REV: 65.91), suggesting a predominant use of defense mechanisms such as altruism and idealization to cope with distress. **Conclusions**: In this selected sample of adults referred for psychological evaluation for suspected or documented workplace victimization, participants showed a clinically relevant psychological burden, including depressive symptoms, somatic concerns, Anger–Hostility, fatigue, reduced vigor, and specific defensive patterns. Given the cross-sectional design, small sample size, and absence of a control group, these findings should be interpreted as preliminary and cannot establish causality or the specificity of this profile to workplace victimization.

## 1. Introduction

Workplace victimization syndrome is a collection of psychological, physical, and behavioral symptoms that emerge in response to prolonged and systematic episodes of workplace violence [1]. Workplace victimization, defined as a form of psychological violence perpetrated by colleagues or superiors against an individual, can have severe consequences on the mental and physical health of the victim [2]. In the scientific literature, workplace mobbing is conceptualized as a form of psychosocial violence that often lacks overt or explicitly aggressive manifestations. Instead, it typically unfolds through subtle, chronic, and systematic behaviors, including persistent denigration, psychological pressure, erosion of professional credibility, social image attacks, exclusion from professional networks, and various forms of ostracism [1,2]. As a reaction, victims of mobbing can develop psychiatric symptoms, including anxiety, chronic stress, depression, sleep disturbances, irritability, anger, and diminished self-esteem. These symptoms are frequently accompanied by somatic complaints and functional impairments, including reduced occupational performance and social withdrawal [3]. From a diagnostic perspective, workplace victimization syndrome is not currently recognized as a standalone diagnostic entity in major diagnostic manuals such as the DSM-5 (Diagnostic and Statistical Manual of Mental Disorders, Fifth Edition) [4] or the ICD-11 (International Classification of Diseases, 11th Revision) [5]. However, the clinical manifestations observed in individuals exposed to workplace victimization can be framed within established diagnostic domains, including trauma-related disorders (e.g., post-traumatic stress disorder), anxiety disorders (e.g., generalized anxiety disorder), mood disorders (e.g., major depressive disorder), and somatic symptom disorders [6]. In this context, depressive symptomatology is considered within the broader category of mood disorders, rather than as a distinct diagnostic entity.

The literature on the psychological and behavioral consequences of workplace victimization highlights a broad spectrum of negative effects on the mental health and behavior of victims. Numerous studies have documented that workplace victimization is associated with severe psychological consequences such as anxiety, depression, post-traumatic stress disorder (PTSD), sleep disturbances, and a significant reduction in self-esteem [7,8]. Chronic exposure to hostile workplace environments is associated with persistent stress activation, which may contribute to emotional dysregulation, cognitive difficulties, and increased vulnerability to mood disorders [9,10]. Behaviorally, victims may withdraw from social interactions and show increased absenteeism, along with impaired concentration and reduced productivity [11,12,13]. These findings highlight the pervasive impact of workplace victimization on both individual functioning and occupational outcomes [14].

Taken together, these findings support the need for a multidimensional assessment approach that considers the interaction between different domains of psychological functioning. Accordingly, the present study integrates the MMPI-2 (Minnesota Multiphasic Personality Inventory-2), POMS (Profile of Mood States), and DMI (Defence Mechanism Inventory) to capture complementary aspects of personality traits, mood states, and defense mechanisms, providing a more comprehensive characterization of the psychological impact of workplace victimization.

Victims often experience a progressive deterioration in emotional well-being, with symptoms of anxiety, depression, and chronic stress [15]. To cope with such conditions, individuals may develop various defense mechanisms [16,17,18], including rationalization and denial, aimed at minimizing or cognitively reframing the experienced abuse [3]. These defensive processes can be interpreted as regulatory strategies that attempt to maintain psychological stability under conditions of chronic interpersonal stress.

Workplace victimization can also affect personality functioning [19]. Prolonged exposure to hostile environments may alter self-perception and interpersonal trust, leading to increased suspiciousness, emotional vulnerability, or maladaptive interpersonal styles. Evidence suggests that such experiences may exacerbate pre-existing traits or contribute to the development of dysfunctional personality patterns [20,21].

Despite extensive research on the psychological consequences of workplace victimization, personality traits, mood states, and defense mechanisms have largely been examined in isolation, and their interaction remains insufficiently understood. From a neuropsychological perspective, workplace victimization may be conceptualized as a chronic stress condition associated with alterations in emotional regulation, cognitive processing, and adaptive coping systems. Within this framework, personality characteristics, mood disturbances, and defense mechanisms can be viewed as interconnected components of psychological functioning that dynamically shape individual responses to prolonged stress exposure. A more integrated investigation of these domains may provide deeper insight into how individuals process and regulate chronic workplace stress, offering clinically relevant implications for understanding the mechanisms underlying psychological distress and maladaptive adjustment, as well as informing both assessment and intervention strategies.

In this context, a more structured investigation of how these domains interact may help to better define the psychological profile associated with workplace victimization and guide the research questions of the present study. Indeed, the present study aims to examine the relationships between workplace victimization, personality traits, mood states, and defense mechanisms, providing preliminary evidence on their multidimensional interaction in individuals exposed to such conditions. In particular, these domains were assessed at the time of evaluation, reflecting the current psychological functioning associated with workplace victimization rather than pre-existing conditions.

To address this gap, a multidimensional assessment approach was adopted, combining the Minnesota Multiphasic Personality Inventory-2 (MMPI-2), the Profile of Mood States (POMS), and the Defence Mechanism Inventory (DMI), which capture complementary aspects of psychological functioning. Specifically, the MMPI-2 was used to assess personality traits and psychopathological features, the POMS to evaluate current mood states and emotional distress, and the DMI to explore defence mechanisms and coping strategies, thereby allowing a more integrated characterization of psychological functioning in individuals exposed to workplace victimization.

## 2. Materials and Methods

### 2.1. Procedure

The recruitment process involved occupational physicians and court-appointed psychologists (CTU) as experts in evaluation and questionnaire administration in medico-legal settings, who adhered to a research protocol and signed agreements for the management of sensitive data in accordance with Italian legislation. Data were collected between January 2022 and December 2023 in private clinical and forensic settings located in different Italian cities, including Messina, Rome, and Milan. The participants were individually assessed in person, using paper-and-pencil methods, within the context of medico-legal consultation for suspected or documented workplace victimization. Workplace victimization was defined according to established criteria in the literature [1] as repeated and systematic exposure to negative acts over time.

Ethical considerations included data confidentiality, voluntary participation with the option to withdraw without consequences, and ensuring that participants were fully informed about the purpose of the study and the management of their data. All information collected in this research was processed in compliance with Italian personal data protection regulations (Legislative Decree 196/2003) and Article 9 of the Italian Psychologists’ Code of Ethics. Personal data were managed with the highest confidentiality and used exclusively for research purposes. Participants had the right to exercise their rights under Legislative Decree 101/2018, which adapts the Code on personal data protection to EU Regulation 2016/679, at any time. The study was conducted in accordance with the Declaration of Helsinki and approved by the Institutional Review Board of the Polish Society of Disaster Medicine (protocol code 13.01.2023. IRB, 3 January 2023), given the international collaboration of the research team.

### 2.2. Participants

The study included 33 adults referred for psychological evaluation in the context of suspected or documented workplace victimization. Inclusion criteria were: (i) Workplace victimization was defined as repeated exposure to negative acts (e.g., verbal abuse, exclusion, or harassment) occurring at least monthly over a minimum of six months, within the work context, and associated with subjective psychological distress [1], (ii) referral for clinical or forensic psychological assessment, and (iii) ability to complete the psychometric evaluation, assessed at recruitment based on language proficiency and cognitive functioning. A total of 35 individuals were initially recruited; however, 2 participants were excluded because they were unable to complete the assessment, resulting in a final sample of 33 participants. Demographic characteristics of the sample are reported in the Section 3.

### 2.3. Outcomes Measures

All assessments were administered by a trained clinical psychologist with expertise in psychodiagnostic evaluation, following standardized administration procedures. To capture complementary dimensions of psychological functioning related to chronic stress exposure, three validated instruments were used, targeting personality traits, mood states, and defense mechanisms. The assessment battery included the Minnesota Multiphasic Personality Inventory-2 (MMPI-2) for personality and psychopathological features, the Profile of Mood States (POMS) for emotional and affective states, and the Defense Mechanism Inventory (DMI) for coping strategies and defense mechanisms (see Table 1).

### 2.4. Statistical Analysis

A descriptive statistical analysis was performed for all variables. Continuous variables were expressed as mean ± standard deviation (SD), while categorical variables were reported as frequencies and percentages. All psychometric data were scored according to standardized procedures based on Italian normative data, and raw scores were converted into T-scores (mean = 50, SD = 10). Scores ≥ 65 were considered clinically significant. The distribution of the data was assessed using the Shapiro–Wilk test. Based on data distribution, Pearson’s correlation coefficients were used for normally distributed variables, while Spearman’s rank correlation coefficients were applied for non-normally distributed data. Correlation analyses were conducted to explore the relationships between personality traits, mood states, and defense mechanisms, with the aim of identifying patterns of multidimensional psychological functioning associated with workplace victimization. All statistical analyses were performed using SPSS software version 27.0 (IBM Corp., Armonk, NY, USA). A two-tailed significance level of *p* < 0.05 was adopted.

## 3. Results

### 3.1. Demographic Characteristics

The sample consisted of 33 participants, including 14 men (42.4%) and 19 women (57.6%), recruited from different professional sectors such as healthcare, education, the armed forces, and small-to-medium enterprises. Regarding educational level, 1 participant (3.0%) had completed primary school, 6 (18.2%) middle school, 16 (48.5%) high school, and 10 (30.3%) held a university degree. With respect to marital status, 17 participants (51.5%) were married, 9 (27.3%) were single, 2 (6.1%) were divorced or separated, 1 (3.0%) was widowed, and 2 (6.1%) were cohabiting; data were missing for 2 participants (6.1%).

### 3.2. Psychological Profiles

The psychological assessment results are presented below, including personality, mood, and defense mechanisms.

#### 3.2.1. MMPI-2 Personality Profile

The MMPI-2 profile showed generally valid response patterns, with L (54.00) within the normal range and a moderately elevated F scale (62.94), suggesting psychological distress. The K scale (46.24) was slightly below average, indicating reduced defensive functioning. Clinically significant elevations (T ≥ 65) were observed in Hypochondriasis (Hs: 72.00), Depression (D: 70.21), Hysteria (Hy: 67.61), and Paranoia (Pa: 68.76), indicating prominent somatic concerns, depressive symptoms, and suspiciousness. Moderate elevations were found in Psychasthenia (Pt: 59.30) and Schizophrenia (Sc: 63.76), suggesting anxiety and cognitive-perceptual disturbances. Other scales, including Psychopathic Deviate (Pd: 57.52), Masculinity-Femininity (Mf: 51.79), Hypomania (Ma: 58.09), and Social Introversion (Si: 55.36), were within or slightly above the average range.

**Figure 1:**
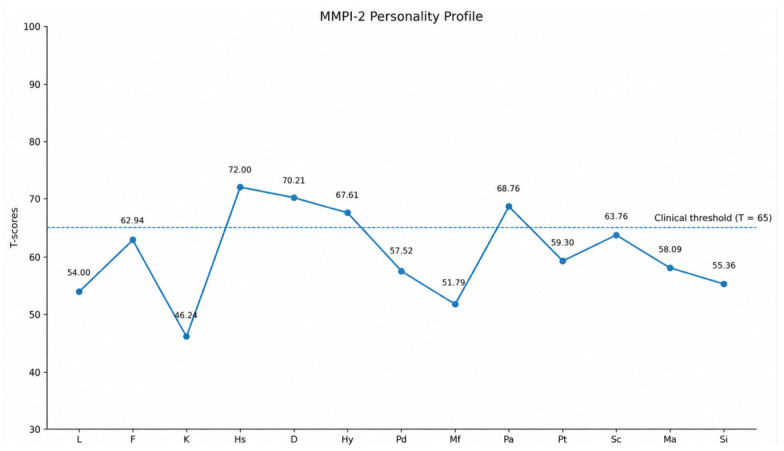
MMPI-2 personality profile of individuals exposed to workplace victimization (N = 33). T-scores are reported for each clinical and validity scale. The dashed line indicates the clinical threshold (T ≥ 65). Legend. L = Lie; F = Frequency; K = Defensiveness; Hs = Hypochondriasis; D = Depression; Hy = Hysteria; Pd = Psychopathic Deviate; Mf = Masculinity-Femininity; Pa = Paranoia; Pt = Psychasthenia; Sc = Schizophrenia; Ma = Hypomania; Si = Social Introversion.

Overall, the MMPI-2 profile indicates a pattern of psychological distress characterized by depressive symptoms, somatization, and paranoid traits (see Figure 1).

#### 3.2.2. POMS Mood Profile

The POMS profile showed elevated levels of mood disturbance across several domains. Clinically relevant elevations were observed in Depression–Dejection (D: 68.21), Tension–Anxiety (T: 65.06), Anger–Hostility (A: 68.15), and Fatigue–Inertia (F: 65.24), indicating increased emotional distress and physical exhaustion. In contrast, Vigor–Activity (V: 43.18) was notably reduced, suggesting diminished energy and vitality. Confusion-Bewilderment (C: 57.94) was within or slightly above the normal range, indicating moderate levels of anxiety and cognitive difficulties.

**Figure 2:**
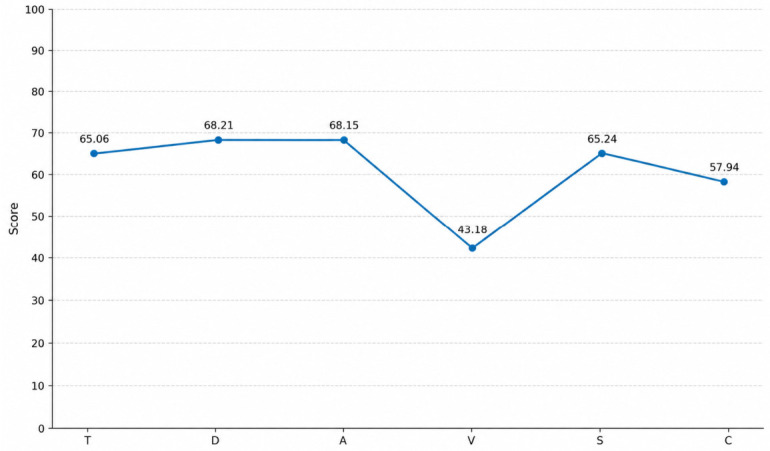
Mood Profile of Victims (N = 33). Legend. T = Tension–Anxiety; D.1 = Depression–Dejection; A = Anger–Hostility; V = Vigor–Activity; S = Fatigue–Inertia; C = Confusion-Bewilderment.

Overall, the POMS profile reflects a pattern of increased negative affect (depression, anger, fatigue) and reduced positive activation (vigor) (see Figure 2).

#### 3.2.3. Defense Mechanisms (DMI)

The DMI profile showed distinct patterns in defensive functioning. The most prominent finding was a marked elevation in Reversal (REV: 65.91), indicating a predominant use of altruistic and idealizing defense mechanisms. Other defense mechanisms were within the average range, including Projection (PRO: 49.16), Principalization (PRN: 52.53), and Turning Against Self (TAS: 50.94). In contrast, Turning Against Object (TAO: 36.06) was notably reduced, suggesting limited outward expression of aggression.

**Figure 3:**
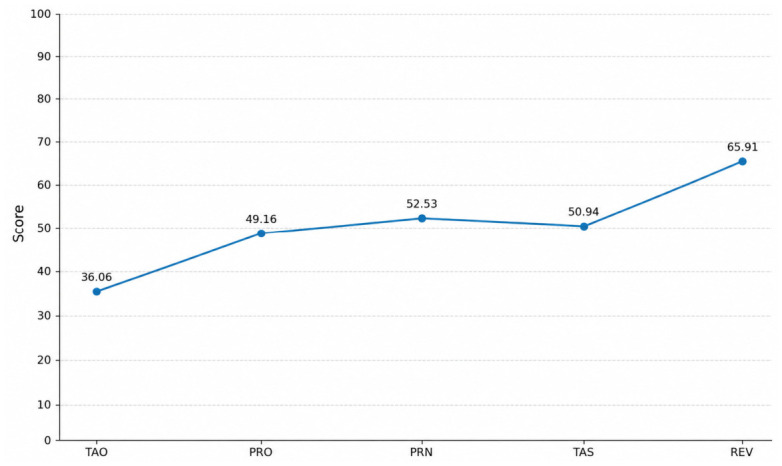
Defense Mechanisms Profile (N = 33). Legend. TAO = Turning Against Object; PRO = Projection; PRN = Principalization; TAS = Turning Against Self; REV = Reversal.

Overall, the profile indicates a tendency to regulate distress through internalized and adaptive-like defense strategies, rather than externalizing responses (see Figure 3).

#### 3.2.4. Correlation Analysis Between Psychological Measures

**Figure 4:**
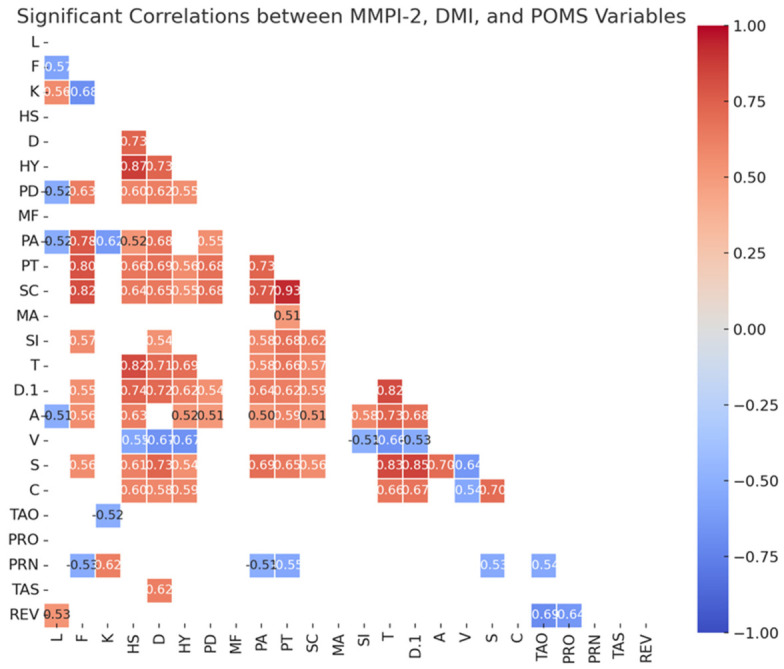
Correlations between personality profile, mood profile, and defense mechanisms. Legend. MMPI-2 Scales: L = Lie; F = Frequency; K = Defensiveness; HS = Hypochondriasis; D = Depression; HY = Hysteria; PD = Psychopathic Deviate; MF = Masculinity-Femininity; PA = Paranoia; PT = Psychasthenia; SC = Schizophrenia; MA = Hypomania; SI = Social Introversion. POMS Scales: T = Tension–Anxiety; D.1 = Depression–Dejection; A = Anger–Hostility; V = Vigor–Activity; S = Fatigue–Inertia; C = Confusion-Bewilderment. DMI Scales: TAO = Turning Against Object; PRO = Projection; PRN = Principalization; TAS = Turning Against Self; REV = Reversal.

Correlation analyses revealed significant associations among personality traits, mood states, and defense mechanisms. Figure 4 displays the correlation heatmap illustrating the significant relationships among variables from the three psychological instruments: MMPI-2, DMI, and POMS. Each row and column corresponds to a specific scale (e.g., L, F, K, clinical scales such as D, HY, PD, and others like TAO, PRO, TAS), and only statistically significant correlations are shown. The color scale ranges from blue (−1.00), indicating strong negative correlations, to red (+1.00), indicating strong positive correlations, with lighter tones representing weaker associations. The correlation matrix reveals a highly coherent pattern in which most MMPI-2 clinical scales (such as Depression, Hysteria, Psychopathic Deviate, Paranoia, Psychasthenia, Schizophrenia, and Social Introversion) are strongly and positively interrelated, indicating the presence of a general psychological distress factor where increases in one symptom dimension tend to co-occur with increases in others. This cluster is further reinforced by substantial positive correlations with anxiety, stress, and confusion-related variables (A, S, C), suggesting that emotional distress and psychopathology form a tightly connected network. In contrast, validity scales (L, F, K) and energy/vigor (V) show a different pattern, often correlating negatively with clinical symptoms, meaning that higher defensiveness or greater vigor tends to be associated with lower reported distress. Notably, vigor exhibits consistent negative relationships with anxiety, stress, and confusion, reflecting the typical inverse association between psychological well-being and activation. Additionally, some defense mechanisms (e.g., PRN) display negative correlations with both distress and validity indicators, suggesting a potentially protective or avoidant function (see Figure 4).

## 4. Discussion

Compared with prior literature, which has predominantly relied on single-instrument or unidimensional approaches, the present study adopts a multidimensional framework that integrates personality traits, current mood states, and defense mechanisms, thereby offering a more comprehensive understanding of psychological functioning in individuals exposed to workplace victimization. While previous research consistently documents associations between victimization and elevated levels of depression, anxiety, and general distress, these relationships are often examined in isolation, resulting in more fragmented and less robust patterns of association. In contrast, the current findings show a coherent pattern of correlations across MMPI-2, POMS, and DMI variables, suggesting the presence of a unified and interconnected distress system in which trait-based psychopathology and state-dependent emotional responses are closely intertwined. This pattern may be consistent with the experience of chronic occupational stress in individuals referred to psychological evaluation in the context of workplace victimization. However, given the cross-sectional design, it is not possible to determine whether these associations reflect pre-existing vulnerabilities, consequences of workplace victimization, or their interaction. The inclusion of defense mechanisms nevertheless provides clinically relevant information on possible regulatory strategies involved in psychological distress, although these findings should be interpreted as preliminary.

The MMPI-2 profile revealed clinically significant elevations in Hypochondriasis (Hs: 72.00), Depression (D: 70.21), Hysteria (Hy: 67.61), and Paranoia (Pa: 68.76), suggesting the presence of somatic complaints, emotional distress, and increased suspiciousness. These findings are consistent with studies by Einarsen et al. [7] and Matthiesen & Einarsen [22], which report an increased vulnerability to depressive and somatoform symptoms in victims of workplace victimization.

Similarly, the POMS results showed elevated negative affect (depression, anger, fatigue) and reduced vigor, reflecting a condition of emotional exhaustion and reduced psychological activation. This pattern aligns with the observations of Niedhammer et al. [9], who highlighted the association between workplace victimization and elevated levels of anxiety, depression, and fatigue.

A key finding of the present study concerns defense mechanisms. The marked elevation in Reversal (REV: 65.91) suggests that individuals tend to rely on altruistic and idealizing strategies to cope with workplace victimization. While such mechanisms may appear adaptive, they may also reflect a tendency to internally regulate distress while avoiding direct confrontation, potentially contributing to the persistence of adverse conditions. This interpretation is consistent with the theoretical observations of Leymann [1], who was one of the first to describe, in a case study, the use of maladaptive coping strategies in victims of prolonged workplace stress.

The correlation analyses provide exploratory evidence of associations between personality-related and mood-related dimensions. Strong associations between MMPI-2 and POMS scales, particularly in depression- and anxiety-related domains, suggest that psychological distress may be expressed across both personality and current mood dimensions. In particular, the correlation between MMPI-2 Depression and POMS Depression–Dejection (r = 0.72) is consistent with previous findings by Balducci et al. [23], supporting the convergence between personality-based depressive features and affective distress. However, these associations should be interpreted cautiously and should not be considered evidence of causal or mechanistic relationships.

Similarly, the association between somatic symptoms and anxiety (MMPI-2 Hypochondriasis and POMS Tension–Anxiety; r = 0.82) is in line with previous findings by Niedhammer et al. [9], suggesting that chronic stress in workplace victimization is often accompanied by somatic manifestations.

Regarding defense mechanisms, the negative correlations between Reversal and MMPI-2 Paranoia and Schizophrenia (r = −0.38; r = −0.35) may suggest that individuals with higher psychopathological burden report lower use of adaptive-like defensive strategies, such as altruism and idealization. Similarly, the negative association between Reversal and depressive mood (r = −0.36) may indicate a relationship between defensive functioning and emotional distress. In line with Leymann’s theoretical observations on the psychological consequences of prolonged workplace victimization [1], these findings may suggest that defensive strategies represent part of the individual’s attempt to regulate distress under chronic occupational stress. However, given the cross-sectional nature of the data, it is not possible to determine whether Reversal has a protective function, reflects a coping style, or represents individual differences in psychological adjustment. Therefore, this finding should be considered preliminary and hypothesis-generating, requiring confirmation in larger and longitudinal studies.

Taken together, these findings suggest that workplace victimization may be associated with a complex interaction between emotional dysregulation, somatic distress, and coping processes, rather than isolated psychological symptoms. This multidimensional perspective has potential clinical implications, suggesting the need for interventions targeting not only mood symptoms but also maladaptive coping patterns and personality-related features/vulnerabilities. Nevertheless, these results should be interpreted cautiously and primarily used to guide future research and clinical hypotheses, rather than to draw definitive conclusions about the specificity or directionality of the observed associations.

### Study Limitations, Strengths, and Future Directions

Several limitations should be considered when interpreting the present findings. First, the relatively small sample size may limit the generalizability of the results, and findings should therefore be interpreted with caution. Second, the cross-sectional design precludes any causal inference regarding the relationships between workplace victimization, personality traits, mood states, and defense mechanisms. In addition, the absence of a control group does not allow the determination of whether the observed psychological profiles are specific to individuals exposed to workplace victimization. Furthermore, the identification of workplace victimization was based on clinical referral and self-reported experiences, which may be subject to reporting bias. The use of self-report psychometric instruments may have also introduced response bias and social desirability effects. Finally, the recruitment procedure, involving occupational physicians and court-appointed expert psychologists, may have contributed to the inclusion of individuals with more severe or clinically complex conditions, potentially influencing the observed psychological profiles.

Despite these limitations, the present study has several strengths. In particular, it adopts a multidimensional approach integrating personality traits, mood states, and defense mechanisms, providing a comprehensive characterization of psychological functioning in individuals exposed to workplace victimization. This integrative perspective represents a relevant contribution to the literature, where these domains have often been investigated separately.

Future research should address these limitations by including larger and more homogeneous samples, as well as longitudinal and controlled study designs, to better clarify the temporal and causal relationships underlying workplace victimization. In addition, the use of multimethod assessment approaches, integrating psychometric, behavioural, and clinical data, would allow for a more comprehensive understanding of the phenomenon and support the development of targeted clinical and organizational interventions.

## 5. Conclusions

The present study provides preliminary evidence of associations between workplace victimization and psychological functioning, suggesting a potential involvement of defense mechanisms in modulating emotional distress. In particular, the analysis of DMI profiles suggests that maladaptive and adaptive-like coping strategies coexist, potentially informing clinical assessment and intervention planning aimed at promoting more functional psychological regulation [24,25].

Overall, these findings are consistent with previous evidence on the negative psychological impact of workplace victimization, while adding an exploratory multidimensional perspective that integrates personality traits, mood states, and defense mechanisms. However, due to the small sample size, cross-sectional design, and absence of a control group, the results should be interpreted with caution and cannot establish causal relationships or determine whether the observed profile is specific to workplace victimization [26].

These findings underscore the importance of timely, targeted clinical interventions and the implementation of effective organizational strategies to prevent and address workplace victimization [27]. Consistent with previous research by Einarsen et al. [7], Niedhammer et al. [9], and Leymann [1], the present study highlights the need for further investigations to better clarify the psychological processes and patterns of association involved, and to develop more effective, evidence-based intervention approaches [28,29]. Future studies should include larger samples, longitudinal designs, and appropriate control groups to clarify the directionality and specificity of these associations and to support the development of more targeted clinical and organizational interventions.

## Figures and Tables

**Table 1:** Overview of psychometric instruments and scoring characteristics.

Instrument	Domain Assessed	Key Dimensions	Scoring/Interpretation
MMPI-2	Personality and psychopathology	Clinical scales (e.g., Depression, Paranoia, Hypochondriasis), Validity scales (L, F, K)	T-scores (Mean = 50, SD = 10); scores ≥ 65 indicate clinically relevant elevations
POMS	Mood states	Tension–Anxiety; Depression–Dejection; Anger–Hostility; Vigor–Activity; Fatigue–Inertia; Confusion-Bewilderment	T-scores; higher scores indicate greater mood disturbance (except Vigor, where lower scores indicate reduced vitality)
DMI	Defense mechanisms	TAO, PRO, TAS, REV, PRN	Standardized scores; higher values indicate greater use of the corresponding defense mechanism

Abbreviations: MMPI-2 = Minnesota Multiphasic Personality Inventory-2; POMS = Profile of Mood States; DMI = Defense Mechanism Inventory; L = Lie; F = Frequency; K = Defensiveness; TAO = Turning Against Object; PRO = Projection; TAS = Turning Against Self; REV = Reversal; PRN = Principalization.

## Data Availability

The data supporting the findings of this study are not publicly available due to privacy and ethical restrictions but are available from the corresponding author upon reasonable request.

## Abbreviations

The following abbreviations are used in this manuscript:

MMPI-2	Minnesota Multiphasic Personality Inventory-2
POMS	Profile of Mood States
DMI	Defense Mechanism Inventory
MMPI-2 scales:	
L	Lie
F	Frequency
K	Defensiveness
Hs	Hypochondriasis
D	Depression
Hy	Hysteria
Pd	Psychopathic Deviate
Mf	Masculinity–Femininity
Pa	Paranoia
Pt	Psychasthenia
Sc	Schizophrenia
Ma	Hypomania
Si	Social Introversion
POMS scales:	
T	Tension–Anxiety
D	Depression–Dejection
A	Anger–Hostility
V	Vigor–Activity
S (or F)	Fatigue–Inertia
C	Confusion-Bewilderment
DMI scales:	
TAO	Turning Against Object
PRO	Projection
PRN	Principalization
TAS	Turning Against Self
REV	Reversal
